# Traditional Uighur Medicine Karapxa decoction, inhibits liver xanthine oxidase and reduces serum uric acid concentrations in hyperuricemic mice and scavenges free radicals *in vitro*

**DOI:** 10.1186/s12906-015-0644-1

**Published:** 2015-04-25

**Authors:** Nurmuhammat Amat, Anwar Umar, Parida Hoxur, Mihrigul Anaydulla, Guzalnur Imam, Ranagul Aziz, Halmurat Upur, Anake Kijjoa, Nicholas Moore

**Affiliations:** Traditional Uighur Medicine Institute, Xinjiang Medical University, 393 Medical University Road, Urumqi, Xinjiang 830011 China; Department of Pharmacology - Université de Bordeaux, F-33076 Bordeaux Cedex, France; Department of Pharmacology, Xinjiang Medical University, 830011-Urumqi, Xinjiang, People’s Republic of China; Neurology Department, Xinjiang Medical University Affiliation of Traditional Chinese Medicine Hospital, 116 Huanghe Road, Urumqi, Xinjiang 83000 China; Instituto de Ciências Biomédicas de Abel Salazar (ICBAS), Universidade do Porto, Rua de Jorge Viterbo Ferreira 228, 40-50-313 Porto, Portugal

**Keywords:** Karapxa decoction, Serum uric acid levels, Xanthine oxidase activities, Antioxidant, Potassium oxonate, Traditional Uyghur medicine

## Abstract

**Background:**

Karapxa decoction (KD) is a Traditional Uighur Medicine used for hepatitis, cholecystitis, gastralgia, oedema, gout and arthralgia. Because of its purported effect in gout, its effects were tested in hyperuricemic mice models induced by yeast extract paste or potassium oxonate, as well as its capacity to scavenge free radicals *in vitro*.

**Methods:**

Hyperuricemia was induced in mice by yeast extract paste or potassium oxonate. KD was given orally for 14 days at 200, 400 and 800 mg/kg/day, with Allopurinol 10 mg/kg/day as positive control. Serum uric acid (UA), and liver xanthine oxidase activity (XO) were measured. Scavenging activity of KD on 1, 1-diphenyl-2-picrylhydrazyl radicals (DPP•), nitric oxide (•NO), superoxide (O_2_•-), efficiency against lipid peroxidation, and XO inhibition were determined *in vitro*.

**Results:**

KD inhibited liver XO activity and reduced serum uric acid in hyperuricemic mice. KD also showed noticeable antioxidant activity, scavenging free radicals (DPP•, •NO and O_2_•-). It was effective against lipid peroxidation and inhibited XO *in vitro*.

**Conclusions:**

This study supports the traditional use of Karapxa decoction to treat hyperuricemia and gout.

## Background

Hyperuricemia and gout are metabolic disorders associated with abnormal amounts of uric acid in the body and uric acid crystals deposition or mobilisation in joints [1]. Hyperuricemia is considered a risk factor for gout, cardiovascular and many other diseases [2]. Uric acid, which is poorly soluble and deposits in articular and renal tissues, is the result of purine metabolism, [3] and especially of xanthine by xanthine oxidase (XO). XO inhibitors such as allopurinol are available to block the final step in uric acid synthesis, reducing the production of uric acid [4]. Control of XO is a key factor in the prevention and treatment of uric acid-related diseases [5,6]. Gout is mostly manifest as painful swelling of digital joints, and can be quite incapacitating. Being both highly painful and visible, it is a prime target of traditional therapy. However gout is but the most visible aspect of uric acid excess: hyperuricemia and uric acid tissue deposition are associated with chronic inflammation, and increased risk of cardiovascular diseases, diabetes mellitus, decreased cognition in the elderly, among others. Decreasing uric acid production may therefore have further benefits than just relieving the painful symptoms of gout [7].

The Traditional Uighur Medicine (TUM) herbal formula, *Karapxa decoction* (KD), composed of seven herbal ingredients (Table 1), including seed, leaves or roots of celery, chicory, fennel and dodders, has long been used for gout and arthralgia in addition to other symptoms such as Hepatic coldness,adiposis hepatica, jaundice, hepatitis, cholecystitis, gastralgia [8]. KD is recorded in the State Pharmacopoeia of People’s Republic of China in the Uighur Medicine volume. The seeds and roots of *Cichorium glandulosum* Boiss. et Huet (Chicory) serve as an important ingredient in KD. Previous studies have shown that extracts of *Cichorium glandulosum* Boiss. et Huet decrease serum uric acid and triglyceride concentrations in animal models [8-10], and may also decrease hyperuricemia in hypertriglyceridemia models [11]. Chicory is also commonly cited on websites for “natural” treatment of gout. Other components of KD also have effects, such as the hepatoprotective effect of *Cuscuta chinensis* against liver toxicity of acetaminophen and other drugs [8,12,13]. It is not clear however whether KD can actually reduce serum uric acid levels in hyperuricemia models and inhibit XO activities. The aim of the present study was to evaluate the effects of KD *in vivo* on reduction of serum uric acid level and XO activity in hyperuricemic mice and to measure XO inhibition and free radical scavenging activity *in vitro*.

**Table 1:** **Medicinal plants contained in Karapxa decoction**

**Botanical name**	**Common name**	**Uighur name**	**Family**	**Part used**	**Quantities**
*Apium graveolen* L.	Celery	Karapxa uruki	Umbelliferae	Seed	30 g
*Apium graveolen* L.	Celery	Karapxa yiltizi	Umbelliferae	Root	30 g
*Cuscuta chinensis* Lam.	Dodders	Sirik yogay uruki	Convolvulaceae	Seed	20 g
*Cichorium glandulosum* Boiss. et Huet.	Chicory	Kasin uruki	Compositae	Seed	15 g
*Foeniculum vulgare* Mill	Fennel	Badranji buya yiltizi posti	Umbelliferae	Root	30 g
*Cichorium glandulosum* Boiss. et Huet.	Chicory	Kasin yiltizi	Compositae	Root	15 g

## Methods

### Chemicals

Xanthine and XO were purchased from Sigma (St. Louis, MO, USA). Potassium oxonate was purchased from Aldrich Inc. 2, 2-diphenyl-1-picrylhydrazyl (DPP•), sodium nitroprusside, N-(1-Naphthyl) ethylenediamine dihydrochloride, phenazine methosulfate (PMS), nitroblue tetrazolium (NBT), nicotinamide adenine dinucleotide (NADH), Ascorbic acid (AA) and thiobarbituric acid (TBA) were supplied by Sigma Co. (St Louis, USA). Assay kits for serum Uric Acid (UA) were obtained from Biosino Biotechnology Company Ltd. Assay kits for liver Xanthine oxidase (XO) were obtained from Nanjing Jiancheng Bioengineering Institute. All other chemicals were of analytical grade.

### Plant material

KD is composed of air-dried powdered raw materials (Table 1) that were purchased from Xinjiang Autonomous Region Traditional Uighur Medicine Hospital (Urumqi, China) and authenticated by associate chief pharmacist Anwar Talip. The voucher specimens (NU-110108, NU-100908, NU-110123, NU-110113, NU-110128, NU-100111) have been deposited in the Xinjiang Autonomous Region Traditional Uighur Medicine Hospital (Urumqi, China).

### Preparation of the aqueous extract of KD

According to the recipe of KD recommended by the State Pharmacopoeia of People’s Republic of China, all herbs were cut into pieces, then 1 kg herbs were marinated in 10 L of warm distilled water for 12 hours. The aqueous extract was then prepared by boiling for 30 min. The extract was filtered and concentrated under reduced pressure and temperature (60°C) on a rotary evaporator, dried in vacuum conditions and stored in the refrigerator. The yield of the extract was found to be 21.84%. The powder was suspended in 0.5% sodium carboxymethylcellulose (CMC-Na) solution before use.

### Animals

Kunming mice weighing 18 ± 22 g were obtained from the Experimental Animal Centre of Xinjiang Medical University. The mice were housed in plastic cages at room temperature of 22 ± 1°C under a 12 h light–dark cycle, and provided with rodent chow and water *ad libitum*. All procedures were in strict accordance with the guidelines set of the Good Laboratory Practice centre at Xinjiang Autonomous Region Traditional Uighur Medicine Institute.

All experimental procedures used in the present study were approved by the Ethics Committee of the Xinjiang Medical University which has adopted the guidelines established by the Xinjiang Uighur Autonomous Region on Animal Care and Experimentation.

### Animal model of hyperuricemia in mice

Two different *in vivo* hyperuricemia models were established using yeast-induced and potassium oxonate stimulated mice, with some modifications [14,15]. Yeast contains large amounts of purine and is used to induce hyperuricemia in mice. For yeast-induced hyperuricemic animal model experiments 60 mice were equally divided into 6 groups as shown in Table 2. The normal control group was given 0.5% CMC-Na orally for 14 days. All other groups of mice were given yeast extract paste (30 g/kg) in 0.5% CMC-Na, orally once per day for 14 days. Group 2 was the hyperuricemic animal model control. Groups 3, 4 and 5 were treated with KD (200 mg/kg, 400 mg/kg and 800 mg/kg) by gavage for 14 days. Group 6 were treated with allopurinol 10 mg/kg orally for 14 days.

**Table 2:** **Effect of Karapxa decoction (KD) or Allopurinol (AP) on serum uric acid (UA) and liver xanthine oxidase (XO) activity in yeast extract paste (YEP) and potassium oxonate (PO) models of hyperuricemic mice** ***in vivo***

**Group**	**Dose**	**UA**	**UA inhibition**	**Liver XO**	**Liver XO inhibition**
	**(mg/kg)**	**(μmol/L)**	**(%)**	**(U/per mg protein)**	**(%)**
Normal	--	6.12 ± 2.12	--	1.84 ± 0.15	--
Model (YEP)	--	135.5 ± 29.2^Δ^	--	3.59 ± 0.28^Δ^	--
YEP + KD	200	96.7 ± 27.8**	28.6	3.05 ± 0.21*	15.0
YEP + KD	400	81.8 ± 22.9**	39.6	2.65 ± 0.13*	26.2
YEP + KD	800	65.5 ± 15.9**	51.6	2.15 ± 0.22**	40.1
YEP + AP	10	15.0 ± 3.0**	88.9	1.28 ± 0.25**	64.3
Normal	--	9.53 ± 1.42	--	1.62 ± 0.23	--
Model (PO)	--	243.1 ± 17.2^Δ^	--	3.86 ± 0.31^Δ^	--
PO + KD	200	196.3 ± 22.8**	19.2	3.15 ± 0.31	18.4
PO + KD	400	162.5 ± 21.0**	33.2	2.81 ± 0.13*	27.2
PO + KD	800	135.5 ± 15.6**	44.3	2.65 ± 0.22*	31.4
PO + AP	10	25.0 ± 19.0**	89.7	1.48 ± 0.15**	61.7

KD: Karapxa decoction; YEP, yeast extract paste model; AP, allopurinol, PO: potassium oxonate model. Data represent mean ± S.E.M. of 10 animals.

^Δ^P <0.05 compared to normal control group.

*P < 0.05 compared to model control group.

**P < 0.01 compared to model control group.

The uricase inhibitor potassium oxonate was used to induce hyperuricemia in mice [16]. Sixty mice were equally divided into 6 groups: the normal control was given orally only 0.5% CMC-Na for 14 days. All other mice were injected intraperitoneally with potassium oxonate 250 mg/kg 1 h before drug administration. Group 2 served as hyperuricemic animal model control. Groups 3, 4 and 5 were treated with KD (200 mg/kg, 400 mg/kg and 800 mg/kg) for 14 days. Group 6 was treated with allopurinol 10 mg/kg orally for 14 days.

### Sample collection and measurement of serum UA, liver XO activities

Whole blood samples were collected from mice 1 h after final administration by retro-orbital sinus puncture. The blood was allowed to clot for approximately 1 h at room temperature and then centrifuged at 3500 × g for 5 min to obtain the serum. The serum was stored at −80°C until assayed. Mouse liver was excised, frozen immediately and stored at −80°C until used. Tissue sample was homogenized in 5 vol. of 50 mM ice-cold phosphate buffer (pH 7.5). The homogenate was then centrifuged for 10 min at 1500 × g at 4°C. The lipid layer was carefully removed and the resulting fraction centrifuged further at 10,000 × g for 30 min and the supernatant was used for assays. Serum UA and liver XO was determined using a commercial kit (Nanjing Jiancheng Biochemical Reagent Co) according to the manufacturer’s instructions. Liver XO activities were expressed as nmol/min per mg protein. Protein concentration was determined using a commercial kit (Nanjing Jiancheng Biochemical Reagent Co) according to the manufacturer’s instructions. The supernatant obtained after the last centrifugation was also used for the assays of XO activities.

### Assay of xanthine oxidase activity

The XO activity was assayed spectrophotometrically under aerobic conditions as reported with minor modifications [17]. The assay mixture consisted of 1 ml of test solution, 2.9 ml of phosphate buffer (pH 7.5), and 0.1 ml of enzyme solution (0.01 units/ml in phosphate buffer, pH 7.5), which was prepared immediately before use. After preincubation at 25°C for 15 min, the reaction was initiated by the addition of 2 ml of substrate solution (50 mM xanthine in the same buffer). The assay mixture was incubated at 25°C for 30 min. The reaction was then stopped by adding of 1 ml of 1 N hydrochloric acid and absorption was measured at 290 nm using a UV spectrophotometer. A blank was also prepared in the same way, but the enzyme solution was added to the assay mixture after adding 1 N hydrochloric acid. The assay was done in triplicate. One unit of XO is defined as the amount of enzyme required to produce 1 μmol of uric acid per min at 25°C. Inhibition of the XO activity was measured spectrophotometrically at 290 nm. The percentage of inhibition of XO activity (I%) was calculated as % I = (A-B)-(C-D)/(A-B) × 100 where A is the XO activity without test extract (total uric acid); B, the blank of A without XO; C, the enzyme activity with test extract (residual uric acid); and D, the blank of C without the enzyme.

### Antioxidant activity

#### Assay for DPP•-free radical scavenging activity

DPP• is a stable free radical that accepts an electron or hydrogen radical to become a stable diamagnetic molecule. The model of scavenging the stable DPP• radical is widely used for relatively rapid evaluation of antioxidant activities. The free radical-scavenging activity of the extract was measured in terms of hydrogen donating or radical-scavenging ability using the stable DPP• radical. DPP•'s purple colour shows a characteristic absorption at 517 nm. As antioxidants scavenge the free radical by hydrogen donation, the colour of the DPP• assay solution becomes light yellow resulting in a decrease in absorbance at 517 nm. Assay was performed in a 96-well microplate using the previously described modified method. Different concentrations of test sample and ascorbic acid were prepared in ethanol and 100 μl of the sample solution pipetted into each well and followed by 100 μl of 0.1 mM ethanolic DPP• solution. The reaction mixture was shaken vigorously and incubated at 37°C for 30 min. Absorbance was measured at 517 nm using a microplate reader. The percentage inhibition (%) of the DPP• radical by the samples was calculated using the following equation: % inhibition = (A_C_ – A_S_) /A_C_) × 100, where A_C_ is the absorbance of the control and A_S_ is the absorbance of the sample. The concentration required to scavenge 50% DPP• free radicals was calculated. All determinations were performed in triplicate.

#### Nitric oxide (•NO) radical scavenging assay

In the applied method, at physiological pH spontaneously generated nitric-oxide interacts with oxygen to produce nitrite ions that can be estimated using a Griess reagent. The scavenging activity of KD towards nitric-oxide was evaluated according to a previously described procedure [18]. The reaction mixture (3 ml) containing sodium nitroprusside (10 mM, 2 ml), 0.5 ml phosphate buffer saline (pH 7.4, 0.01 M) and extract or standard solution (0.5 ml) was incubated at 25°C for 150 min. Thereafter, 0.5 ml of the reaction mixture containing nitrite was pipetted and mixed with 1 ml of sulfanilic acid reagent (0.33% sulfanilic acid in 20% glacial acetic acid) and allowed to stand for 5 min for completing diazotization. Then, 1 ml of naphthylethylenediamine dihydrochloride (0.1%) was added, mixed and allowed to stand for 30 min. The absorbance of pink coloured chromophore was measured at 540 nm against the corresponding blank solutions. The IC_50_ value is the concentration of sample required to inhibit 50% of nitric oxide free radical. All tests were carried out in triplicates.

#### Assay for Superoxide anion (O_2_·) scavenging

In the PMS–NADH–NBT system, superoxide anions are derived from dissolved oxygen by the PMS–NADH coupling reaction, which then reduced NBT to a blue coloured formazan. Absorbance is measured at 560 nm. Decrease in absorbance is directly proportional to the antiradical potential of the product tested. Measurement of the superoxide anion scavenging activity of KD was based on the modified method previously reported [19]. Superoxide radicals were generated in phenazine methosulphate (PMS)-nicotinamide adenine dinucleotide (NADH) systems by NADH oxidation and assayed by nitroblue tetrazolium (NBT) reduction. In this experiment, the superoxide radicals were generated in 3 mL of Tris–HCl buffer (16 mM, pH 8.0) containing 0.5 mL of NBT (300 μM) solution, 0.5 mL NADH (936 μM) solution, and 0.5 mL of KD solution at different doses. The reaction was started by adding 0.5 mL of PMS solution (120 μM) to the mixtures. The reaction mixture was incubated at 25°C for 5 min, and the absorbance at 560 nm measured against blank samples. All tests were performed in triplicate and results averaged. The percentage of inhibition was determined by comparing the results of control and test samples.

#### Lipid peroxidation assay

To test the *in vitro* inhibition of lipid peroxidation by the extracts, lipid peroxidation induced by Fe^2+^/ascorbate system in mouse liver homogenate was used and thiobarbituric acid-reactive substances (TBARS) were measured with some modifications [20]. The reaction mixture contained mouse liver homogenate 0.1 ml (25%, w/v) in Tris–HCl buffer (20 mM, pH 7.0), KCl (150 mM), FeSO_4_ · 6H_2_O (0.8 mM), ascorbic acid (0.3 mM) and various concentrations of the extract in a final volume of 0.5 ml and was incubated for 1 h at 37°C. The incubated reaction mixture (0.4 ml) was treated with sodium dodecyl sulphate (0.2 ml, 8%) and thiobarbituric acid (1.5 ml, 20%). The total volume was then made up to 4 ml by adding distilled water and kept in a water bath maintained at 100°C for 1 h. After cooling, 1 ml of distilled water and 5 ml of n-butanol were added and shaken vigorously to separate the butanol fraction and measure TBARS formed at 535 nm. The percentage of inhibition of lipid peroxide formation was determined by comparing the absorbance of the treated extract and non-treated samples.

#### Statistical analysis

Values are presented as mean ± S.E.M. Analysis of variance (ANOVA) was used to test for differences among treated and controlled groups. Inhibitory concentration 50% (IC_50_) of each sample was calculated by linear regression analysis using SPSS 11.0 software programme.

## Results

### Effect on uric acid

The effects of KD on yeast extract paste (YEP) and uricase inhibitor potassium oxonate (PO) induced hyperuricemia in mice are shown Table 2. YEP and PO caused hyperuricemia in mice, with serum uric acid level increased to 135.5 ± 29.2 μmol/L and 243.1 ± 37.2 μmol/L, after 10 days oral YEP or 2 h after intraperitoneal PO, respectively. Fourteen days treatment with KD at 200, 400, 800 mg/kg effectively opposed the increase of serum UA concentrations in both models. Allopurinol significantly lowered serum uric acid concentrations in experimental animals, to values not different from normal controls.

### Liver XO inhibitory activity

The XO activities in normal groups were 1.84 ± 0.35 and 1.62 ± 0.23 U/per mg protein respectively. The liver XO activities were increased to 3.56 ± 0.28 and 3.86 ± 0.31 U/per mg protein in the model control mice (p < 0.05). KD inhibited YEP-induced XO activity by 15.0%, 26.2%, 40.1% in 200, 400 and 800 mg XD-treated mice, respectively and PO-induced XO activity by 18.9%, 27.2%, 31.4% inhibition for 200, 400 and 800 mg/kg XD groups (p < 0.05 except 200 mg, NS). Allopurinol inhibited mouse model XO by 64.3% and 61.66% at the dose of 10 mg/kg (both p < 0.05), obviously more than KD (Table 2).

### *In vitro* effects

#### Inhibition of xanthine oxidase

**Figure 1:**
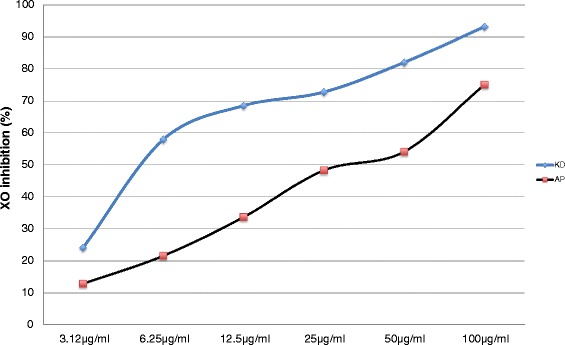
Concentration-response curves for *in vitro* Xanthine Oxidase inhibition (in %) by Xarapxa decoction (XD) or allopurinol (AP) at increasing concentrations.

At a concentration of 250 μg/ml KD, uric acid formation was completely suppressed; the IC_50_ value for KD inhibition was 25.8 μg/ml. Allopurinol had an IC_50_ value of 6.28 μg/ml (Figure 1).

#### DPP• radical scavenging

The results concerning DPP• free radical scavenging effect of KD are indicated in Table 3. KD was a potent DPP• free radical scavenger. This activity was 31.1%, 45.2%, and 65.2%, respectively, for 25, 50, and 100 μg/ml of KD (all p < 0.05). The IC_50_ value was calculated to be 55.6 μg/ml.

**Table 3:** **Effect of increasing concentrations of Karapxa decoction (KD) on radical scavenging with DPP•, Nitric oxide (•NO), Superoxide, and lipid peroxidation models, compared to ascorbic acid or butylated toluene controls**

**Product**	**Concentration (μg/ml)**	**DPP• scavenging (%)**	**•NO scavenging (%)**	**Superoxide scavenging (%)**	**Lipid peroxidation inhibition (%)**
KD	3.13 μg/ml	6.13 ± 1.23	13.3 ± 1.89	4.8 ± 0.4	12.0 ± 2.65
KD	6.25 μg/ml	15.7 ± 1.46	31.3 ± 2.72	8.6 ± 4.23	21.2 ± 3.33
KD	12.50 μg/ml	31.1 ± 3.21	49.1 ± 2.84	23.0 ± 3.69	29.3 ± 2.15
KD	25.0 μg/ml	45.2 ± 2.56	64.2 ± 5.52	33.4 ± 4.12	43.4 ± 4.96
KD	50.0 μg/ml	65.2 ± 3.14	71.5 ± 4.82	49.7 ± 3.88	48.5 ± 4.48
KD	100.0 μg/ml	82.2 ± 3.67	83.3 ± 2.52	69.1 ± 5.19	65.7 ± 4.18
AA	50.0 μg/ml	84.3 ± 4.68	39.2 ± 3.39		
BHT	50.0 μg/ml			21.1 ± 2.43	61.1 ± 2.91

KD: Karapka decoction; AA: Ascorbic acid; BHT: Butylated hydroxytoluene.

#### Nitric oxide (•NO) radical scavenging

The efficiency of KD to scavenge •NO free radical is presented in Table 3. KD was found to possess strong NO scavenging ability, showing concentration-dependent activity (p < 0.05). The IC_50_ value was 31.4 μg/ml.

#### Superoxide anion radical scavenging

As shown in Table 3, KD exhibited significant and concentration dependent O_2_•- scavenging activity (5.8–83.5% inhibition, p < 0.05 for concentrations above 6.25 μg/ml) at 6.25-400 μg/ml, The IC_50_ value was calculated to be 89.3 μg/ml for a scavenging effect at 49.7%.

#### Inhibition of lipid peroxidation

KD displays dose-dependent lipid peroxidation inhibition, and this activity was 24.5%, 34.2%, 48.3%, and 63.5%, for 25 μg/ml, 50 μg/ml, 100 μg/ml, and 200 μg/ml KD (all p < 0.05). The concentration of KD needed for 50% inhibition was found to be 111.3 μg/ml (Table 3).

## Discussion

Karapka decoction had dose-dependent effects on uric acid concentration and xanthine oxidase activity in two different hyperuricemic mice models. In addition, KD had free radical scavenging activities *in vitro*.

Gout and hyperuricemia are increasingly common disorders, reportedly afflicting more than 2 million men and women in the United States alone [21], and is progressing rapidly in China due probably to recent changes in dietary habits [22]. Gout and hyperuricemia are metabolic disorders associated with abnormal uric acid concentrations in the body, resulting in the deposition of urate crystals in the joints and kidneys that lead to inflammation, as well as gouty arthritis and uric acid nephrolithiasis. In addition to an increased risk of hyperuricemia and gout, excess uric acid is also related to cardiovascular disorders, nephrolithiasis, diabetes [7,23-32].

Two major mechanisms have been proposed for hyperucicemia in man, excess production and insufficient metabolisation of uric acid. Yeast extract paste and potassium oxonate were used to mimic both mechanisms: yeast represents excess production of UA, probably the main mechanism in man, and oxonate impairs metabolisation [15,23]. Yeast disturbs normal purine metabolism by increasing xanthine oxidase (XO) activity and generating large quantities of uric acid. This model is similar to human hyperuricemia, which is induced by high-protein diets. Another mouse hyperuricemia model was generated by a single intraperitoneal injection of potassium oxonate 250 mg/kg. Potassium oxonate, a urate oxidase inhibitor, can raise the serum uric acid concentration by inhibiting the decomposition of uric acid by uricase, an enzyme that does not exist in man. Karapxa was found approximately equally effective in both models, but less than allopurinol.

Many bioactive products have been identified from the herbs in the *KD* formula and pharmacological activity of those herbs has been reported. Celery seeds are used in arthritic pain relief, for treating rheumatic conditions and gout [33,34]. Essential oil, fatty acid, flavonoids isolated from *Apium graveolen L*., possesses antibacterial, antioxidative, hepaprotective, anti-tumor, and anti-cardiovascular disease abilities [35,36]. Certain indole-like compounds and indole alkaloids isolated from *A. graveolens* seeds were found to have antioxidant, cyclooxygenase and topoisomerase inhibitory activity [37]. The seeds of *Cuscuta chinensis Lam*. (Convolvulaceae), is a commonly used traditional Chinese herbal medicine used in improving and conditioning the liver and the kidney and also possesses anticancer effects, immunostimulant, antioxidant, and hepatoprotective activities [13,38-41].

The active constituents of *C. chinensis* include flavonol, flavonoids, lignans, quinic acid, and polysaccharide [42-45], which have been suggested to be responsible for the pharmacological activities observed from *C. chinensis* [46,47]. *Cichorium glandulosum* Boiss. et Huet is well known in Uighur medicine use for curing liver diseases, etc. In the Chinese Pharmacopoeia of the People’s Republic of China, “Juju” (*Herba Cichorii* and *Radix Cichorii*) refers to the aerial parts and roots of *Cichorium intybus* L. and *Cichorium glandulosum* Boiss. et Huet. Different parts of these plants have been analysed, mostly for the presence of phenolics and sesquiterpene lactones. The major phenolics include flavonoids, coumarins and caffeic acid derivatives [48].

Many pharmacological studies on the same compounds have been performed earlier, finding anti-diabetic [49], antibacterial [50], hepatoprotective [12] and antioxidant effects. These constituents may be present at different amounts in the KD extracts and it is far from established which constituent(s) are responsible for the effects of the extracts. Further investigations are warranted to identify the active principle(s) of the extracts from KD, responsible for the observed hypouricemic effects.

## Conclusion

The objective of the present study was to test the hypouricemic and antioxidant effects of the KD, which is used in traditional Uighur medicine to treat gout and hyperuricemia. The results suggest that KD at the dosage of 200, 400 and 800 mg/kg has hypouricemic effects in both hyperuricemic mouse models. KD was also found to have inhibitory effects on mouse liver XO activity. Antioxidant activity was evident, through the ability to scavenge several free radicals (DPPH, NO and O_2_^.^) and the effect on lipid peroxidation.

These results, though there is still a need for clinical validation, support the traditional use of KD to prevent or treat hyperuricemia and gout. It was less powerful than allopurinol, but may have the advantages of low cost and high acceptability in countries with limited resources or with a tradition of herbal medicinal treatment of diseases.

## Abbreviations

KD: Karapxa Decoction
XO: Xanthine oxidase
DPPH: 1, 1-diphenyl-2-picrylhydrazyl radicals
PMS: Phenazine methosulfate
NBT: Nitroblue tetrazolium
NADH: Nicotinamide adenine dinucleotide
AA: Ascorbic acid
TBA: Thiobarbituric acid
UA: Uric Acid
NO: Nitric oxide
O_2_: Superoxide
BHT: Butylated hydroxytoluene
